# First report of parasitism by *Eutrichophilus cercolabes* (Phthiraptera: Trichodectidae) on *Coendou spinosus* (Erethizontidae) in Rio Grande do Sul, Brazil – case report

**DOI:** 10.29374/2527-2179.bjvm000823

**Published:** 2023-08-21

**Authors:** Julia Somavilla Lignon, Diego Moscarelli Pinto, Rafael Almeida Fighera, Silvia Gonzalez Monteiro

**Affiliations:** 1 Veterinarian, Laboratório de Parasitologia Veterinária, Departamento de Microbiologia e Parasitologia, Universidade Federal de Santa Maria, Santa Maria, RS, Brazil; 2 Veterinarian, DSc., Departamento de Veterinária Preventiva, Universidade Federal de Pelotas, Pelotas, RS, Brazil; 3 Veterinarian, DSc., Laboratório de Patologia Veterinária, Departamento de Patologia, Universidade Federal de Santa Maria, Santa Maria, RS, Brazil; 4 Veterinarian, DSc., Laboratório de Parasitologia Veterinária, Departamento de Microbiologia e Parasitologia, Universidade Federal de Santa Maria, Santa Maria, RS, Brazil

**Keywords:** arthropods, ectoparasitism, lice, Insecta, artrópodes, ectoparasitismo, piolhos, Insecta

## Abstract

*Coendou spinosus* is a species of rodent popularly known as porcupine, it has a great ability to adapt to different habitats and is found in tropical forests in countries such as Bolivia, Brazil, Venezuela and Guianas. This mammal has already been identified as a reservoir of several pathogenic agents for humans and other animals and has a variety of ectoparasites, endoparasites and hemoparasites little studied and described. Due to this, the objective was to report the parasitism by *Eutrichophilus cercolabes* in *C. spinosus* in the central region of Rio Grande do Sul, southern Brazil. In total, 16 lice were found, one male and 15 females of the species. This is the first report of the parasitism of this Phthiraptera on *C. spinosus* in Rio Grande do Sul. The scarcity of reports on the taxonomy and biotic characteristics, as well as the vector capacity of pathogens of most species of ectoparasites of wild animals, highlights the need for further studies on the distribution of these arthropods in different regions and host species.

## Resumo

*Coendou spinosus* é uma espécie de roedor conhecida popularmente por porco-espinho, possui uma grande capacidade de adaptação a diferentes habitats sendo encontrada em florestas tropicais de países como Bolívia, Brasil, Venezuela e Guianas. Esse mamífero já foi identificado como reservatório de vários agentes patogênicos para o homem e outros animais e possui uma variedade de ectoparasitos, endoparasitos e hemoparasitos pouco estudados e descritos. Devido a isso, objetivou-se relatar o parasitismo por *Eutrichophilus cercolabes* em *C. spinosus* na região central do Rio Grande do Sul, Sul do Brasil. Ao total, foram encontrados 16 piolhos, sendo um macho e 15 fêmeas da espécie. Esse é o primeiro relato do parasitismo deste Phthiraptera em *C. spinosus* no Rio Grande do Sul. A escassez de relatos sobre a taxonomia e características bióticas, bem como a capacidade vetorial de patógenos da maioria das espécies de ectoparasitos de animais silvestres evidencia a necessidade da realização de mais estudos sobre a distribuição desses artrópodes em diferentes regiões e espécies hospedeiras.

## Introduction

*Coendou spinosus* Cuvier, 1823, originally called *C. villosus* (Voss, 2011), is a species of arboreal rodent of the Erethizontidae family, which corresponds to the New World porcupine, commonly known as “ouriço-cacheiro” in Brazil (Caldara Junior & Leite, 2012). The species is found in tropical forests in countries such as Bolivia, Brazil and Venezuela, in addition to the Guianas. It has a nocturnal habit, slow and discreet behavior, being an animal difficult to observe (Voss & Emmons, 1996).

Rodents can be reservoirs of several pathogenic agents, mainly viruses, helminths, bacteria and protozoa (Linardi & Guimarães, 2000). According to the literature, hedgehogs (*Coendou* spp.) can host ectoparasites (*Amblyomma* spp. and *Eutricophilus* spp.), endoparasites (*Prosthenorchis* sp., *Hymenolepis* spp. and *Trichuris* spp.) as well as hemoparasites (*Hepatozoon* sp., *Babesia* spp., *Trypanosoma* spp. and filaria) (Thoisy et al., 2000; Brum et al., 2003; Labruna et al., 2004; Kuniy & Brasileiro, 2006).

Lice infestation in hedgehogs can be high, and this is mainly due to the difficulty in getting rid of the ectoparasites because they have long spines that cover their bodies (Werneck, 1936; Timm & Price, 1994). Timm and Price (1994) recognized 18 species of chewing lice of the genus *Eutrichophilus* parasitizing hedgehogs, which are extremely specific in relation to their hosts (Timm & Price, 1999). They are found only in the New World and in hedgehogs of the Erethizontidae family. According to Werneck (1950), three species can specifically parasitize *C. villosus* (synonym of *C. spinosus*): *Eutrichophilus cercolabes* Mjöberg, 1910, *Eutrichophilus minor* Mjöberg, 1910 and *Eutrichophilus cordiceps* Mjöberg, 1910.

Due to the great adaptability of rodents, some species are frequent in rural and urban areas, often being related to the transfer of pathogenic agents to other animals, such as domestic animals, including humans (Oliveira et al., 2010). In addition, ectoparasites can cause damage to the hosts, as they cause weight loss and also produce lesions that predispose to secondary infections (Monteiro, 2017). In Rio Grande do Sul, mentions of ectoparasites in wild animals are rare. Therefore, the present work aims to report, for the first time, parasitism by *E. cercolabes* on *C. spinosus* in the central region of Rio Grande do Sul, southern Brazil.

## Material and methods

**Figure 1:**
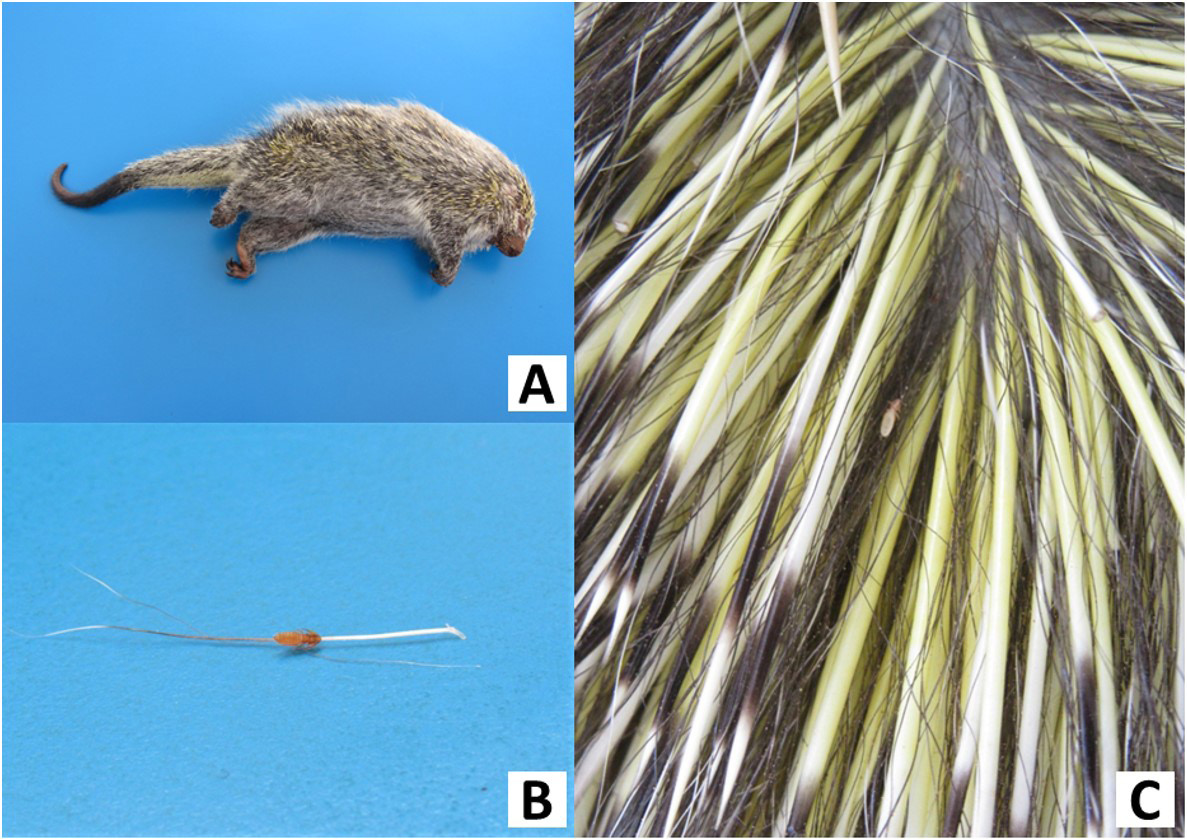
A - *Coendou spinosus*; B - Detail of *Eutrichophilus cercolabes* lice fixed on spine of *C. spinosus*; C- View of *E. cercolabes* lice on *C. spinosus*.

Sixteen specimens of lice stored in 70% alcohol were received at the Veterinary Parasitology Laboratory of the Federal University of Santa Maria for taxonomic classification. The lice were collected during the necropsy of *C. spinosus* specimen, performed at the Laboratory of Animal Pathology at the same institution (Figure 1A). The hedgehog in question was an adult male that was found on the banks of highway RST 287, near the District of Palma, Municipality of Santa Maria, Rio Grande do Sul. It was alive and was brought by people to the University Veterinary Hospital of the Federal University of Santa Maria. He died during the treatment and was immediately sent for necropsy. It was in excellent condition and was necropsied the same day it entered the service. Subsequently, the lice were clarified in phenol-xylene (1:1) and permanently mounted in Canada balsam as described by Monteiro (2017). For the identification, the works of Werneck (1950) and Timm and Price (1994, 1999) were used. The captured images of lice were performed with the ZEN 2® (Blue edition) Carl Zeiss Microscopy program, 2011, 100x and 400x magnification.

## Results and discussion

**Figure 2:**
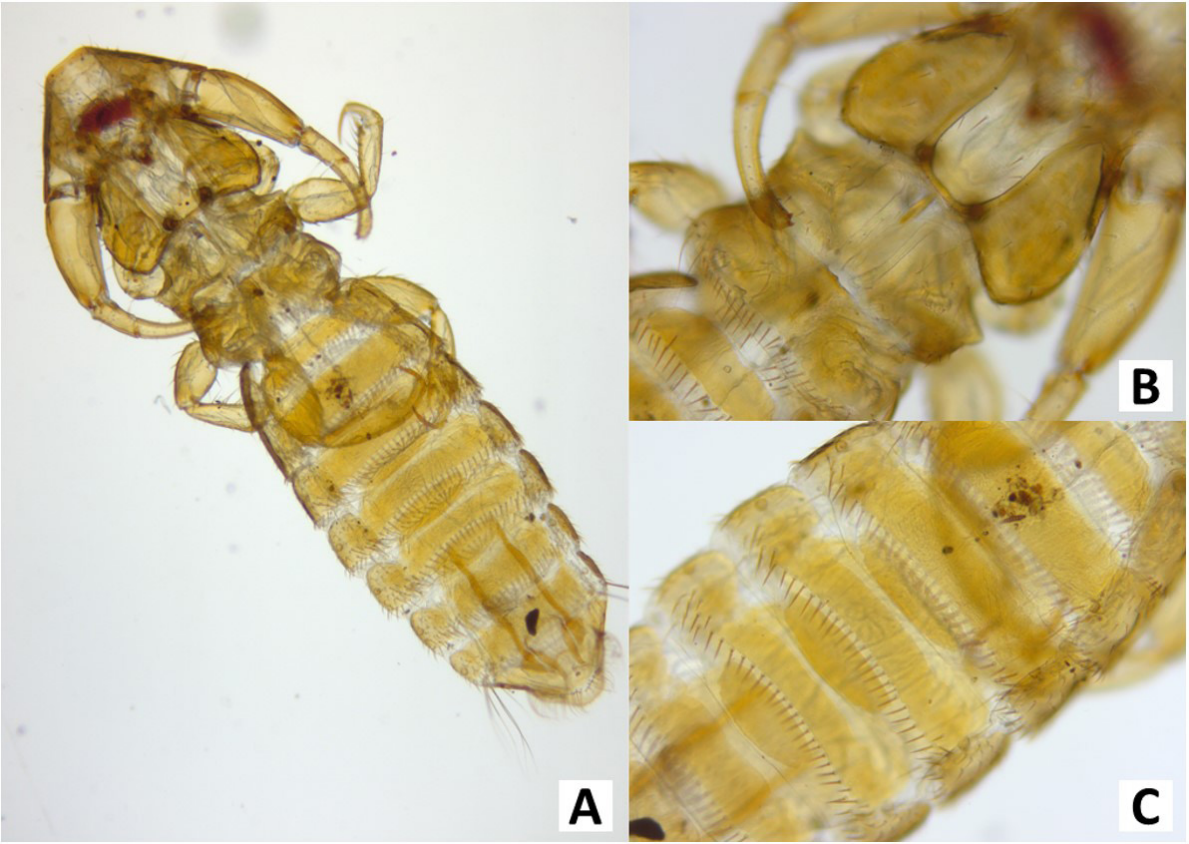
Dorsal view of *Eutrichophilus cercolabes* male. A - Head longer than wide with symmetrical medioanterior margin, flattened to concave and presence of short head bristles. Legs II-III unmodified, similar to leg I but larger. Male terminalia tapered, narrowly rounded; B - Metanotum with a total of 21-29 marginal bristles between the long corner bristles; C - Accessory tergal sclerites and presence of large spiracles.

**Figure 3:**
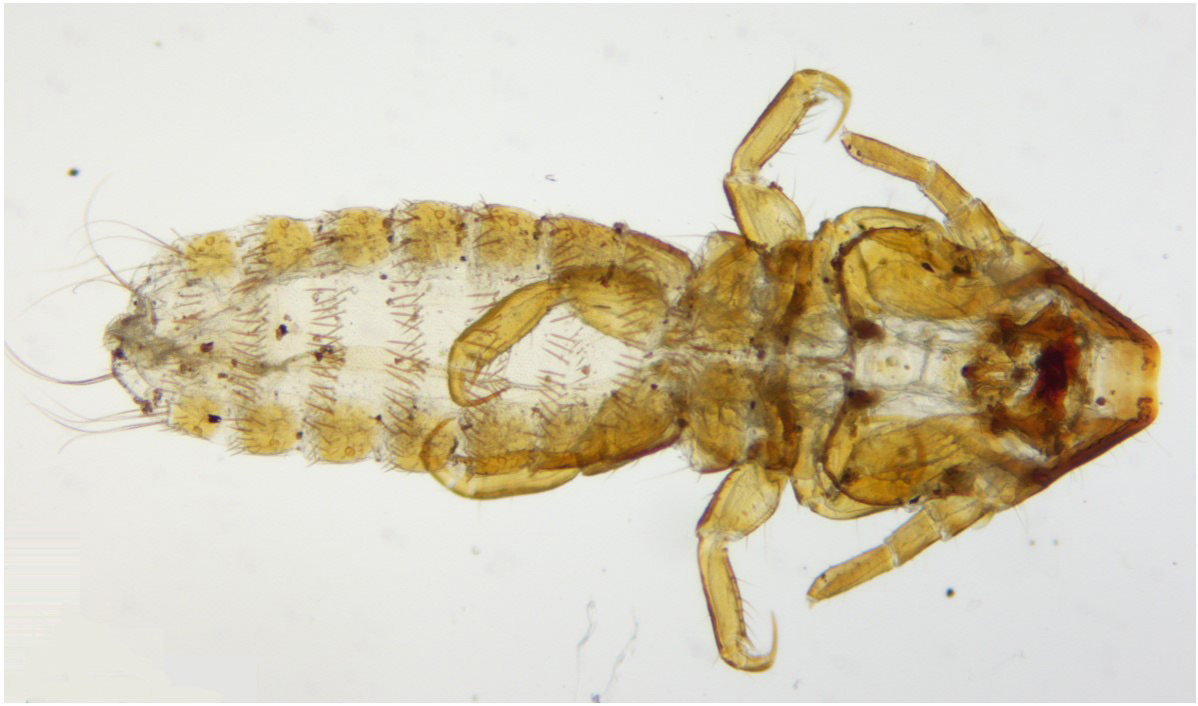
Dorsal view of *Eutrichophilus cercolabes* female. Head longer than it is wide and body without accessory tergal plates. Tergal setae: I, 3-9; II, 25-30; III-VIII, 29-41 and last segment with 19-27 short setae anterior to and mediad of longsetae.

In total, 16 lice of the genus *Eutrichophilus* were identified, one male (Figure 2) and 15 females (Figure 3). According to Werneck (1950), this genus is characterized by presenting a sub-trapezoidal pre-antennary region and the temples strongly projected backwards, having sexual dimorphism by the antennae, an extremely accentuated character. All specimens were identified as *E. cercolabes* (Figures 2-3).

Still according to Werneck (1950), *C. spinosus* is host to *E. cercolabes*, *E. minor* and *E. cordiceps*, the latter being the predominant species in relation to the others and that *E. cercolabes* is always found in smaller number (Amaral, 2008), differently from the present study, in which, *E. cercolabes* was the only species found. Timm and Price (1994) describe the occurrence of males and females in approximately equal numbers in the hosts, unlike the present study, where we observed only one male and 15 females. Amaral (2008), in his study, evaluated the parasitic fauna of rodents and marsupials in the Pedra Branca State Park, located in Rio de Janeiro, Brazil, also reports that the predominant species in *C. spinosus* was *E. cercolabes* (N=75), followed by *E. minor* (N=7), with no specimens of *E. cordiceps* being found.

Timm and Price (1994) cited the identification of E. cercolabes parasitizing C. spinosus from Nova Teotônia, Santa Catarina. The same authors also cite the occurrence of E. cercolabes in C. spinosus in the Colônia de Sta. Cruz, Rio Grande do Sul, currently known as Santa Cruz do Sul. However, this specific locality was associated with the specimens originally described by Mjoberg, which were destroyed during World War II. Weidner (1966) reported that the Hamburg Museum lice used by Mjoberg in 1910 in his dissertation were, without exception, from a zoo animal. Timm and Price (1994), in their review of the genus Eutrichophilus, question the accuracy of this locality, as often with specimens from older zoos, the localities associated with them represent the point from which they were exported from the country, rather than a place of origin. specific catch. As it is a captive animal, it is possible that several individuals were housed or shipped together, thus allowing the transfer of the lice (Timm & Price, 1994). In Rio Grande do Sul, there is still a description of the occurrence of the genus Eutrichophilus parasitizing C. spinosus (Brum et al., 2003), but there was no identification at the species level, just as there is no description and identification of the place where the animals were found.

Therefore, this is the first report of *E. cercolabes* in *C. spinosus* in the Rio Grande do Sul state. Little is known about the taxonomy, biology, ecology, geographic distribution, usual hosts and vector capacity of pathogens of the vast majority of ectoparasite species of wild animals. The scarcity of reports of this nature highlights the need for further studies on the distribution of these arthropods in different regions and host species.

## Conclusion

According to the taxonomic characteristics, the analyzed species is *E. cercolabes*, this being the first report of the parasitism of this Phthiraptera in *C. spinosus* in Rio Grande do Sul.
